# Speech prediction of a listener via EEG-based classification through subject-independent phase dissimilarity model

**DOI:** 10.1038/s41598-025-12135-y

**Published:** 2025-07-18

**Authors:** Alireza Malekmohammadi, Josef P. Rauschecker, Gordon Cheng

**Affiliations:** 1 Institute for Cognitive Systems, Electrical Engineering, Technical University of Munich, 80333 Munich, Germany; 2 Laboratory of Integrative Neuroscience and Cognition, Department of Neuroscience, Georgetown University Medical Center, Washington, DC 20057 USA

**Keywords:** Speech, Phase, EEG, Classification, Auditory system, Computational neuroscience, Sensory processing

## Abstract

This study examines the consistency of *cross-subject* electroencephalography (EEG) phase tracking in response to auditory stimuli via speech classification. Repeated listening to audio induces consistent EEG phase alignments across trials for listeners. If the phase of EEG aligns more closely with acoustics, *cross-subject* EEG phase tracking should also exhibit significant similarity. To test this hypothesis, we propose a generalized subject-independent phase dissimilarity model, which eliminates the requirement for training on individuals. Our proposed model assesses the duration and number of *cross-subject* EEG-phase-alignments, influencing accuracy. EEG responses were recorded from seventeen participants who listened three times to 22 unfamiliar one-minute passages from audiobooks. Our findings demonstrate that the EEG phase is consistent within repeated *cross-subject* trials. Our model achieved an impressive EEG-based classification accuracy of 74.96%. Furthermore, an average of nine distinct phasic templates from different participants is sufficient to effectively train the model, regardless of the duration of EEG phase alignments. Additionally, the duration of EEG-phase-alignments positively correlates with classification accuracy. These results indicate that predicting a listener’s speech is feasible by training the model with phasic templates from other listeners, owing to the consistent *cross-subject* EEG phase alignments with speech acoustics.

## Introduction

Electroencephalography (EEG) is a non-invasive method that measures electrical activity in the brain by placing electrodes on the scalp^1^. EEG signals reflect the summated postsynaptic potentials from large populations of neurons, primarily cortical pyramidal cells, and are characterized by rhythmic fluctuations known as neural oscillations^2,3^. These oscillations occur in distinct frequency bands—namely, delta (1–4 Hz), theta (4–8 Hz), alpha (8–13 Hz), beta (13–30 Hz), and gamma (30–60 Hz)—each of which has been associated with different auditory cognitive and perceptual functions.

In the auditory domain, especially during speech perception, these oscillations are thought to support sensory encoding, temporal integration, acoustic modulation, speech comprehension, and speech perception^4–7^. Listening to spoken languages, such as audiobooks, induces phase entrainment of these neural oscillations—meaning the phase (or timing) of the brain’s rhythmic activity synchronizes with the rhythmic temporal patterns of natural speech^8,9^. In simpler terms, the phase of neural oscillations recorded through EEG reflects how the brain locks onto the timing of acoustic information in speech^10–13^.

To assess how well brain activity synchronizes with external auditory stimuli like speech, one commonly used metric is phase coherence. Phase coherence (also called inter-trial phase coherence or phase-locking value) measures the consistency of the phase of neural oscillations across repeated trials or across different individuals when exposed to the same stimulus. A high phase coherence value indicates that the timing of neural responses is reliably aligned with the stimulus across instances. This temporal alignment is especially prominent in low-frequency oscillations, such as delta and theta bands (< 10 Hz), which are particularly sensitive to the slow rhythmic modulations of natural speech.

Prior studies have shown that amplitude- or power-based measures are often more sensitive to cognitive states such as attention, memory load, or arousal^2^. Notably, a previous study demonstrated that power or amplitude coherence measures did not yield reliable classification performance to discriminate speech stimuli successfully^5^. Unlike amplitude or power-based measures, phase coherence captures the brain’s precise timing relationships with the auditory signal, making it a robust indicator of neural entrainment^7,14–19^. This phase-based consistency enables new possibilities for decoding speech information from brain signals. For example, repeated exposure to the same audiobook produces consistent EEG phase patterns across trials for an individual. Leveraging this observation, researchers have attempted to classify or predict speech stimuli based on phase-based neural responses. However, previous models have shown limitations: they are often subject-specific, require individual training, and typically perform with low classification accuracy^5,20–22^.

This study introduces a novel subject-independent model based on a metric we term cross-subject phase alignment, which measures the similarity in EEG phase responses to a given audiobook across different listeners. The central hypothesis is that if EEG phase aligns strongly with the acoustic structure of speech, then this alignment should generalize across individuals.

The study has two primary goals: (1) to test whether this phase alignment is observable not just within trials of a single subject (cross-trial phase alignment) but also across trials from different subjects (cross-subject phase alignment), and (2) to build a subject-independent classification model using this cross-subject phase similarity.

Our study involved recording EEG signals from 17 participants as they listened to 22 passages of English stories, each lasting between 65 s and 73 s, three times. In this context, we define the “phase” of EEG as the angle-like quantity in degrees or radians measuring the relative position of a waveform at a given time, while “speech” refers to a passage of an audiobook. This study utilized the following methods to predict audiobook preferences based on EEG signal classification:

1. Computing cross-subject phase dissimilarity to extract significant spatial (i.e., electrodes) and spectral (i.e., frequency bins) features,
2. Reproducing a subject-specific dissimilarity model as a benchmark^5^,
3. Developing and evaluating a subject-independent dissimilarity model,
4. Comparing the accuracy of the subject-independent model with the benchmark for different numbers and different durations of *cross-subject* EEG phase alignments,
5. Investigating the relationships between numbers and duration of *cross-subject* EEG phase alignments,
6. Implementing a subject-independent logistic regression classifier, representing an alternative linear machine learning classifier, and
7. Implementing a subject-independent fully connected deep neural network (FCNN), representing an alternative nonlinear deep learning approach.

Compared to previous research, our study introduces three major innovations. First, prior EEG-based speech classification models have predominantly relied on subject-specific training, requiring individual calibration and offering limited generalizability across listeners^5,14,22^. In contrast, our work proposes a subject-independent model that generalizes across participants with fixed hyperparameters for all subjects, by leveraging cross-subject phase alignment, eliminating the need for subject-specific retraining. Second, earlier models often failed to achieve performance significantly above chance^5,20,21^. However, this study introduces a subject-independent model to address the shortcomings of previous models. Third, our work is the first to systematically examine how the number and duration of cross-subject phase templates impact classification accuracy, providing a more scalable and generalizable framework for EEG-based speech decoding.

This study uncovered that the EEG phase modulation of delta (1–4 Hz) and theta (4–8 Hz) bands is consistent across trials of different subjects and can discriminate the 22 one-minute stories with close to 75% accuracy with the subject-independent model. Moreover, it shows that nine phasic templates from other subjects (i.e., cross-subject EEG phase alignment) are sufficient to overcome the subject-specific model. Finally, it demonstrates that, on average, the minimum number of templates required to train the subject-independent model is independent of the duration of audiobooks.

## Results

### EEG phase pattern discriminates acoustics in delta and theta bands

**Fig. 1:**
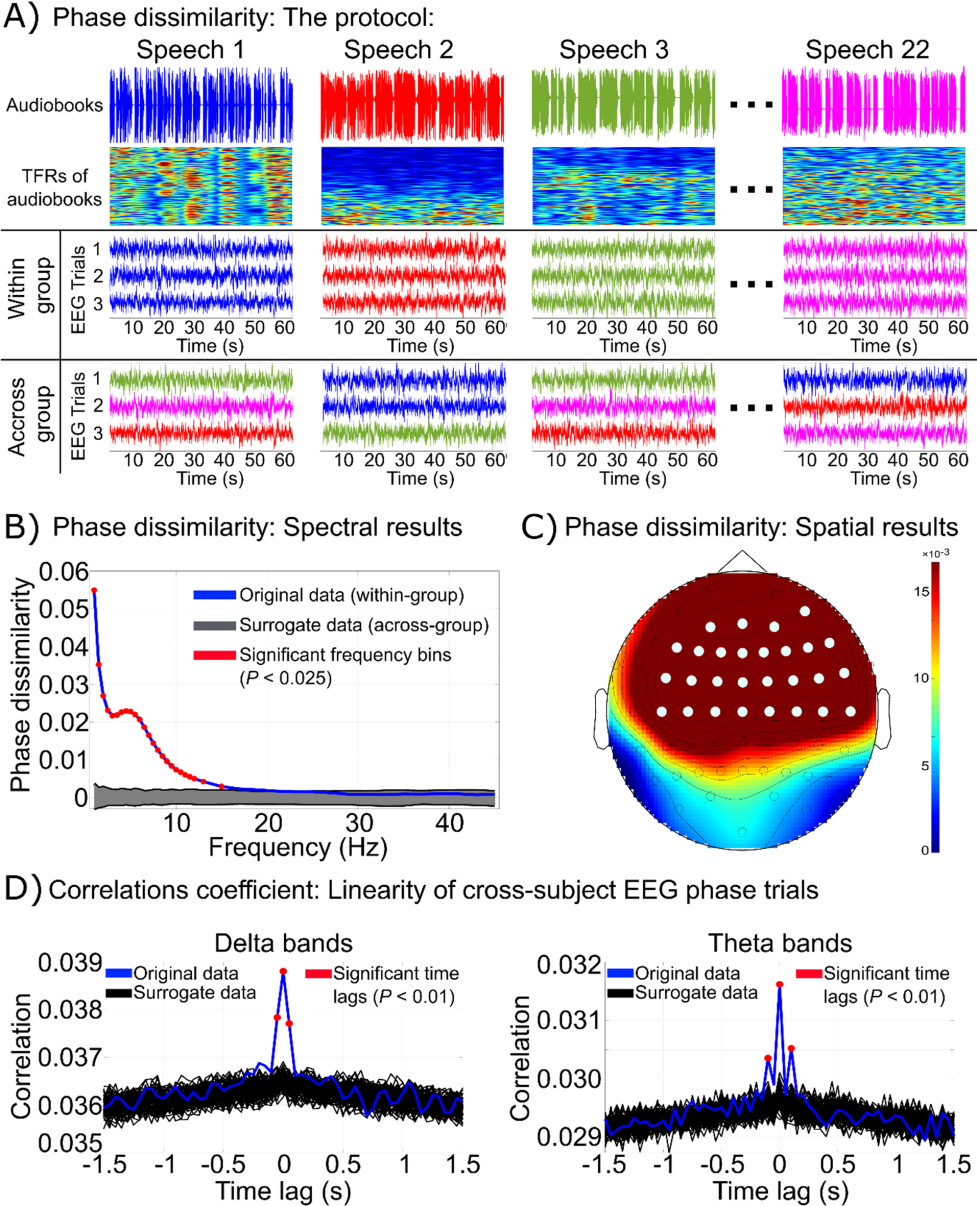
Dissimilarity phase and cross-correlation coefficient. **A**: Indicating the details of the dissimilarity phase protocol for audiobooks and corresponding EEG data, aimed at investigating the consistency of cross-subject phase tracking in EEG signals. Responses to the same audiobook are represented by the same color (within-group), while a random selection of responses across audiobooks is defined as across-group (mixed colors). **B**: The phase dissimilarity is plotted as a function of frequency [1–45] Hz, demonstrating significant phase tracking from 1 Hz to 8 Hz. Within-group phase dissimilarity is shown in blue, across-group phase dissimilarity in gray, and the significant frequency bins [1–8] Hz (Z-score > 11.30, *P* < 0.001) in red. **C**: The topo-plot of 33 significant electrodes (Z-score > 24, *P* < 0.025) with high phase dissimilarity values: Fp1, Fp2, AF7, AF3, AFz, AF4, AF8, F5, F3, F1, Fz, F2, F4, F6, F8, FT7, FC5, FC3, FC1, FCz, FC2, FC4, FC6, FT8, C5, C3, C1, Cz, C2, C4, C6, T8, and CP2. **D**: Cross-correlation coefficient across EEG phase trials of all subjects to evaluate the linearity of cross-subject EEG phase alignments. The significant time lags are shown in red (Z-score > 13.50, *P* < 0.01).

This paper proposed the phase-dissimilarity algorithm^5^ to investigate the potential for distinguishing between 22 audiobooks based on the corresponding phase patterns of EEG responses (see Fig. 1A). Phase dissimilarity—defined as the difference in cross-subject phase coherence between within-group and across-group trials—was computed across various frequency bins from 1 Hz to 45 Hz (see Fig. 1B). The results demonstrate that EEG phase patterns within the group significantly differentiate audiobooks when the within-group phase coherence is notably higher than across the group. Figure 1B reveals that within-group EEG phase coherence is significantly (Z-score > 11.30, *P* < 0.001) greater than across-group phase coherence in low frequencies, especially in the 1–8 Hz frequency range. No significant differences were observed at frequencies above 15 Hz.

Additionally, Fig. 1C illustrates the topography of averaged phase dissimilarity across delta (1–4 Hz) and theta (4–8 Hz) bands, identifying the most informative EEG electrodes for classifying one-minute responses to each audiobook. Notably, the bilateral frontal and frontocentral electrodes (i.e., Fp1, Fp2, AF7, AF3, AFz, AF4, AF8, F5, F3, F1, Fz, F2, F4, F6, F8, FT7, FC5, FC3, FC1, FCz, FC2, FC4, FC6, FT8, C5, C3, C1, Cz, C2, C4, C6, T8, and CP2) exhibited distinctive phase patterns (Z-score > 24, *P* < 0.025). Electrodes highlighted in stronger red tones correspond to higher delta and theta phase dissimilarity, indicating their relevance for audiobook classification.

Furthermore, Fig. 1D presents the cross-correlation coefficients computed across EEG phase trials from all subjects, used to evaluate the linear relationship of cross-subject EEG phase alignment over time lags ranging from − 1.5 s to + 1.5 s for both delta and theta frequency bands. The original correlations are shown in blue, surrogate correlations in black, and statistically significant time lags are highlighted in red (Z-score > 13.50, *P* < 0.01). As shown in Fig. 1D, the correlation coefficients at a time lag of zero seconds are significantly higher than those derived from the surrogate data for both frequency bands (*p* < 0.01). These results provide evidence of significant linear alignment among EEG phase responses across subjects when exposed to the same stimulus, supporting the suitability of linear models, such as nearest-template classification, for capturing these cross-subject patterns.

### The performance for EEG classifications of 22 classes

**Fig. 2:**
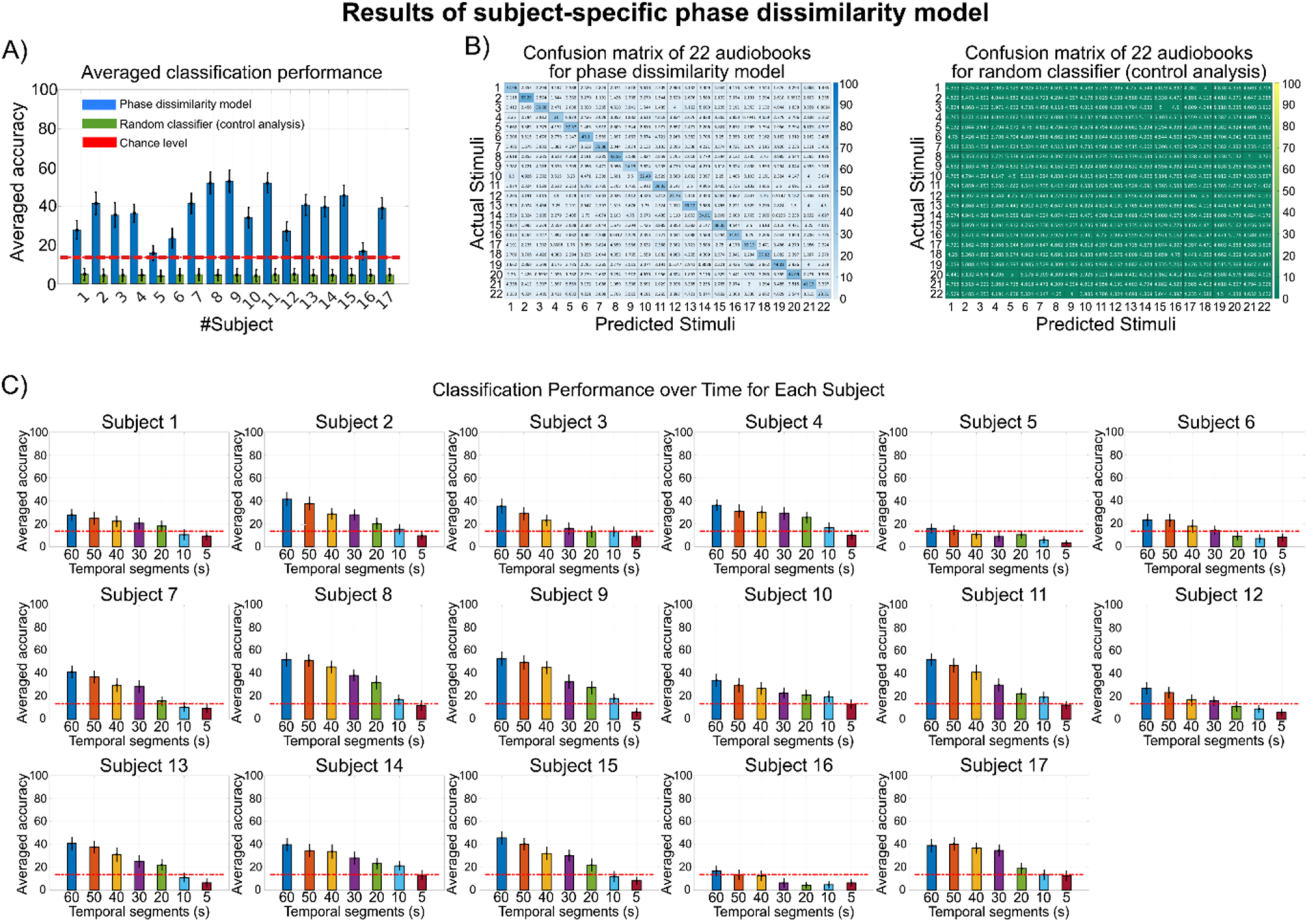
Results of the subject-specific phase dissimilarity model. **A**: The average accuracy for each subject for the subject-specific model and random classifier that uses a single trial of the same subject as a template (grand average accuracy of the model is 36.57%). **B**: The confusion matrices of the model and random classifier summarize predictions for all audiobooks (i.e., stimuli). The best model’s classification belongs to audiobook #2, with an accuracy of 53.29%. **C**: Evaluating classification performance across different duration lengths for each subject indicates a decrement in accuracy when the length of the audiobooks decreases. At least 60 s of audiobooks are needed to classify EEG signals above the chance level.

**Fig. 3:**
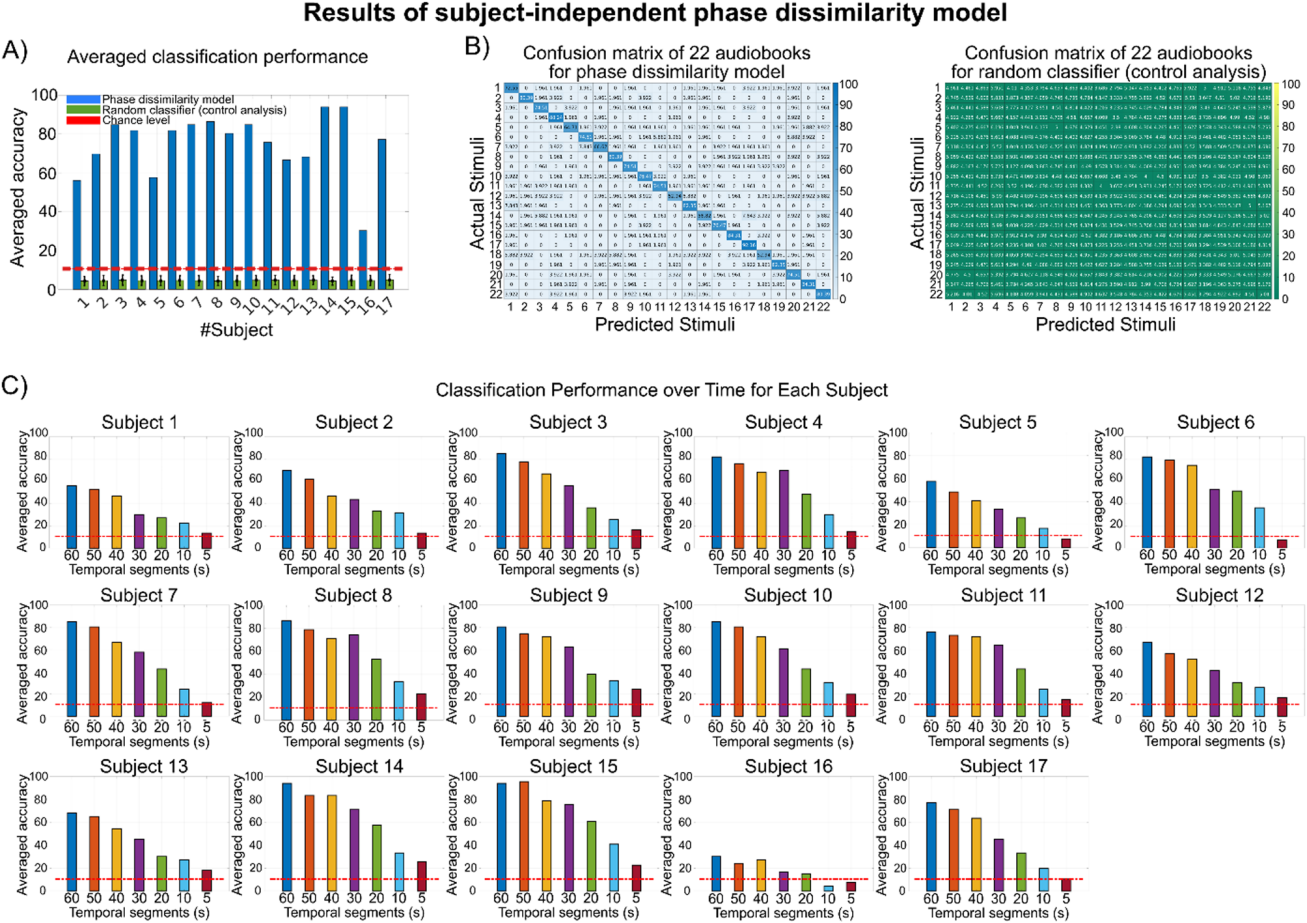
Results of the subject-independent phase dissimilarity model. **A**: The average accuracy for each subject for the subject-independent model and random classifier that utilizes trials from other subjects as templates, excluding trials from the same subject (grand average accuracy is 74.96%). **B**: The confusion matrices of the model and random classifier summarize predictions for all audiobooks (i.e., stimuli). The best model’s classification belongs to audiobook #17, with an accuracy of 92.16%. **C**: Evaluating classification performance across different duration lengths for each subject indicates a decrement in accuracy when the length of the audiobooks decreases. At least 20 s of audiobooks are needed to classify EEG signals above the chance level.

In Figs. 2A and 3A, the average accuracy across all 22 audiobooks is presented for each subject and each model. The subject-specific phase dissimilarity model achieves a mean accuracy of 36.57%, while the subject-independent model achieves a substantially higher mean accuracy of 74.96%. For all participants, the classification accuracies in both models exceed the performance of a random classifier and the chance-level threshold determined using the binomial inverse cumulative distribution function.

The subject-specific model exhibits variable performance across individuals, ranging from a low of 15.89% for subject 5 to a high of 52.90% for subject 9. In contrast, the subject-independent model demonstrates consistently higher accuracy, with the lowest at 30.30% for subject 16 and the highest at 93.94% for both subject 14 and subject 15.

We acknowledge that the subject-independent model benefits from having a larger and more averaged template set (16 subjects × 3 repetitions = 48 trials) compared to the subject-specific model, which uses only one template per class. This discrepancy in template quantity may partly explain the improved performance of the subject-independent model due to reduced noise and higher generalizability. To address this concern, the following section explores how the number of templates affects the performance of the subject-independent model. In particular, we quantify how many templates are required before it begins to outperform the subject-specific model.

In Figs. 2B and 3B, the confusion matrices summarize the classification performance of both models and the corresponding random classifiers for each audiobook. The subject-specific model achieved higher classification accuracy for audiobooks 2, 6, 8, 19, 20, and 21, each with at least 40% accuracy. In contrast, the subject-independent model yielded higher classification accuracy for audiobooks 2, 4, 8, 13, 16, 17, 19, 21, and 22, each with at least 80% accuracy. Notably, audiobook 2 attained the highest accuracy in the subject-specific model at 53.29%, while audiobook 17 achieved the highest accuracy in the subject-independent model at 92.16%.

### Classification performance over the duration of cross-subject EEG phase alignments

In the previous sections, the study utilized EEG responses to predict the audiobooks being listened to. Specifically, the phase of the delta and theta bands was analyzed while the participants listened to one minute of each audiobook. This section investigates how the duration of EEG phase alignment influences classification performance. To examine this, temporal segments of the EEG responses (i.e., the first 50 s, first 40 s, first 30 s, first 20 s, first 10 s, and first 5 s) were extracted, and the same classification models were applied. As shown in Figs. 2C and 3C, all models demonstrate a gradual decline in classification accuracy as the segment length decreases, indicating a positive correlation between classification performance and EEG phase alignment duration. Figure 2C shows that at least 60 s of EEG data (*P* < 0.01) are needed to achieve above-chance accuracy for all subjects using the subject-specific model. Conversely, Fig. 3C reveals that the subject-independent model reaches above-chance accuracy with just 20 s of EEG data (*P* < 0.01), highlighting its robust and superior performance compared to the subject-specific model.

### Classification performance over the number of cross-subject EEG phase alignments

**Fig. 4:**
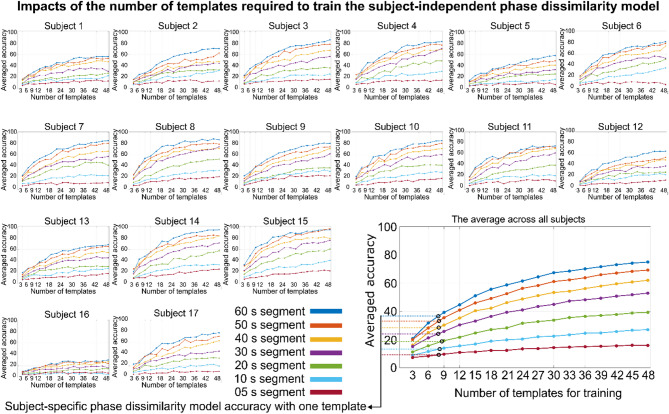
Impacts of the number of cross-subject phase alignments required to train the subject-independent phase dissimilarity model. The average accuracy is shown per subject and segment for different numbers of templates needed to train the subject-independent model. On average, a minimum of nine templates is required to overcome the accuracy of the subject-specific model for each segment.

In the preceding section, we presented individual outcomes of a subject-independent model trained using data from 16 other participants. Each participant listened to the audiobooks three times, yielding a total of 48 EEG phasic templates (i.e., 16 subjects × 3 trials) used to train the subject-independent model. In contrast, the subject-specific model was trained using only one template per subject. In this section, we investigate how many templates are required for the subject-independent model to outperform the subject-specific model. Here, a “template” refers to a single EEG trial. Figure 4 presents both individual and overall classification accuracy for different audiobook durations as the number of templates increases from 3 (templates from one randomly selected subject) to 48 (all available templates). As shown in Fig. 4, accuracy improves with the number of templates, particularly for longer durations. Notably, the subject-independent model achieves higher accuracy than the subject-specific model when trained with at least nine templates (See Table 1 for quantified results). Interestingly, the minimum number of templates required to surpass the averaged subject-specific performance appears to be independent of audiobook duration (See Table 1 for quantified results).

**Table 1:** Comparison of average classification accuracy (ACC) across all subjects between subject-specific model and subject-independent models using varying numbers of EEG phase templates for all duration (see Fig. 4).

Duration of EEG templates	ACC of the subject-specific model	ACC of the subject-independent model with different numbers of templates					
	1 template	3 temp.	6 temp.	9 temp.	12 temp.	…	48 temp.
5 s	9.2%	7.4%	8.3%	**9.7**%	**11.0**%	**…**	**15.9**%
10 s	13.3%	9.0%	11.6%	**14.0**%	**15.6**%	**…**	**27.0**%
20 s	18.8%	11.3%	15.6%	**19.3**%	**21.7**%	**…**	**39.3**%
30 s	24.1%	15.1%	21.2%	**25.8**%	**30.4**%	**…**	**52.9**%
40 s	28.5%	16.1%	25.0%	**30.3**%	**35.3**%	**…**	**62.0**%
50 s	33.2%	20.1%	28.2%	**35.5**%	**40.9**%	**…**	**69.3**%
60 s	36.6%	20.7%	31.8%	**39.3**%	**44.7**%	**…**	**75.0**%

**Fig. 5:**
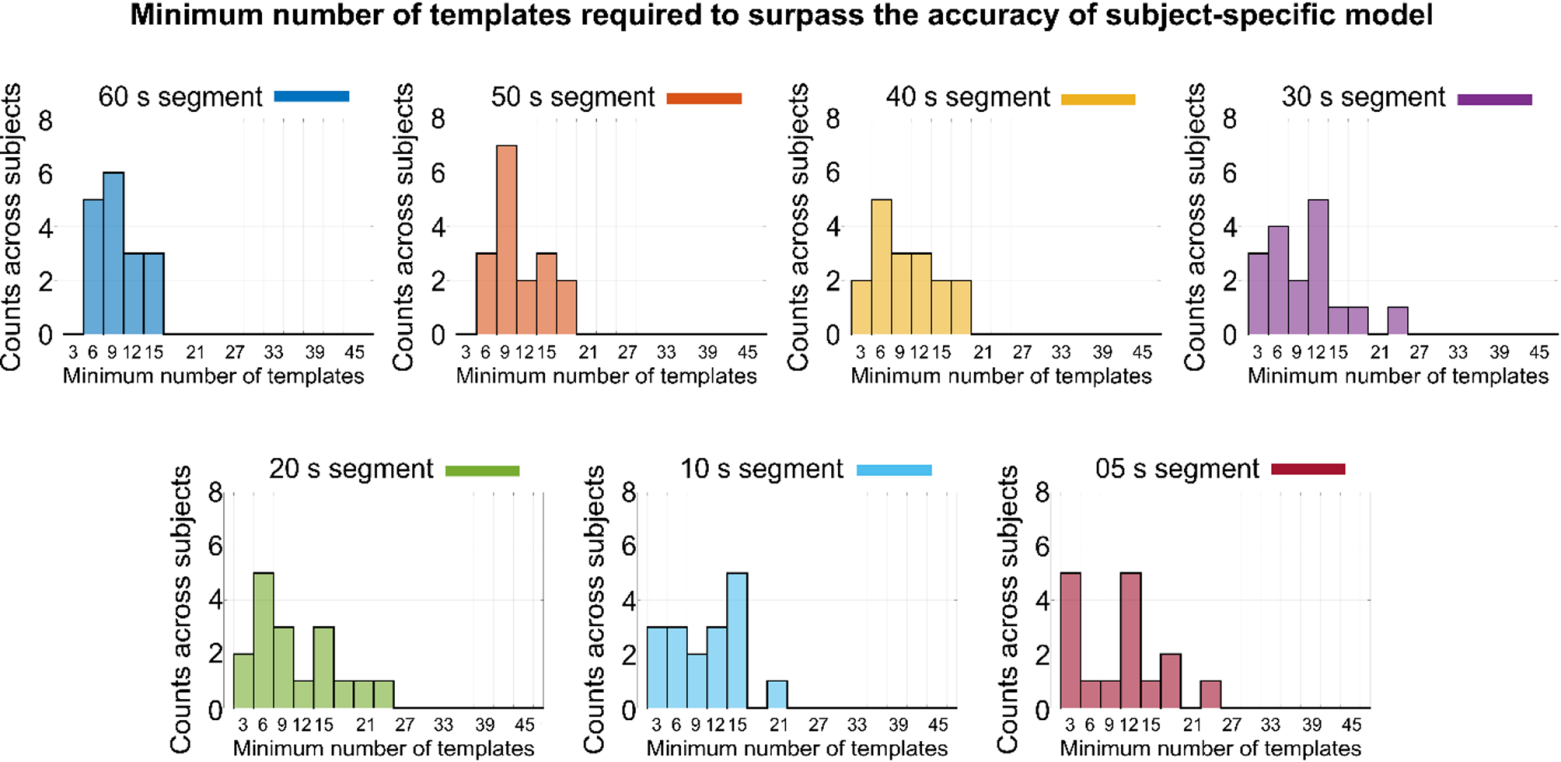
Histogram plots related to the minimum number of cross-subject EEG phase alignments required to overcome the accuracy of the subject-specific model across subjects. The number of templates increases as the length of segments decreases. At least 18 templates are required if the length of the segment is longer than 30 s, while the minimum number of templates increases (a maximum of 24 templates) if the length of the segment is equal to or less than 30 s.

The histograms in Fig. 5 illustrate the minimum number of templates required to train a subject-independent model that surpasses the accuracy of a subject-specific model across all subjects for all segment durations. The data show that as the duration of EEG phase alignment decreases, a greater number of templates are needed to outperform the subject-specific model. Notably, 18 templates are sufficient for all subjects when the EEG segment duration is 40 s or longer. However, for durations shorter than 40 s, the subject-independent model may require more than 18 templates (a maximum of 27 templates) to exceed subject-specific accuracy for certain individuals.

### Classification performance over subject-independent logistic regression and FCNN models

**Fig. 6:**
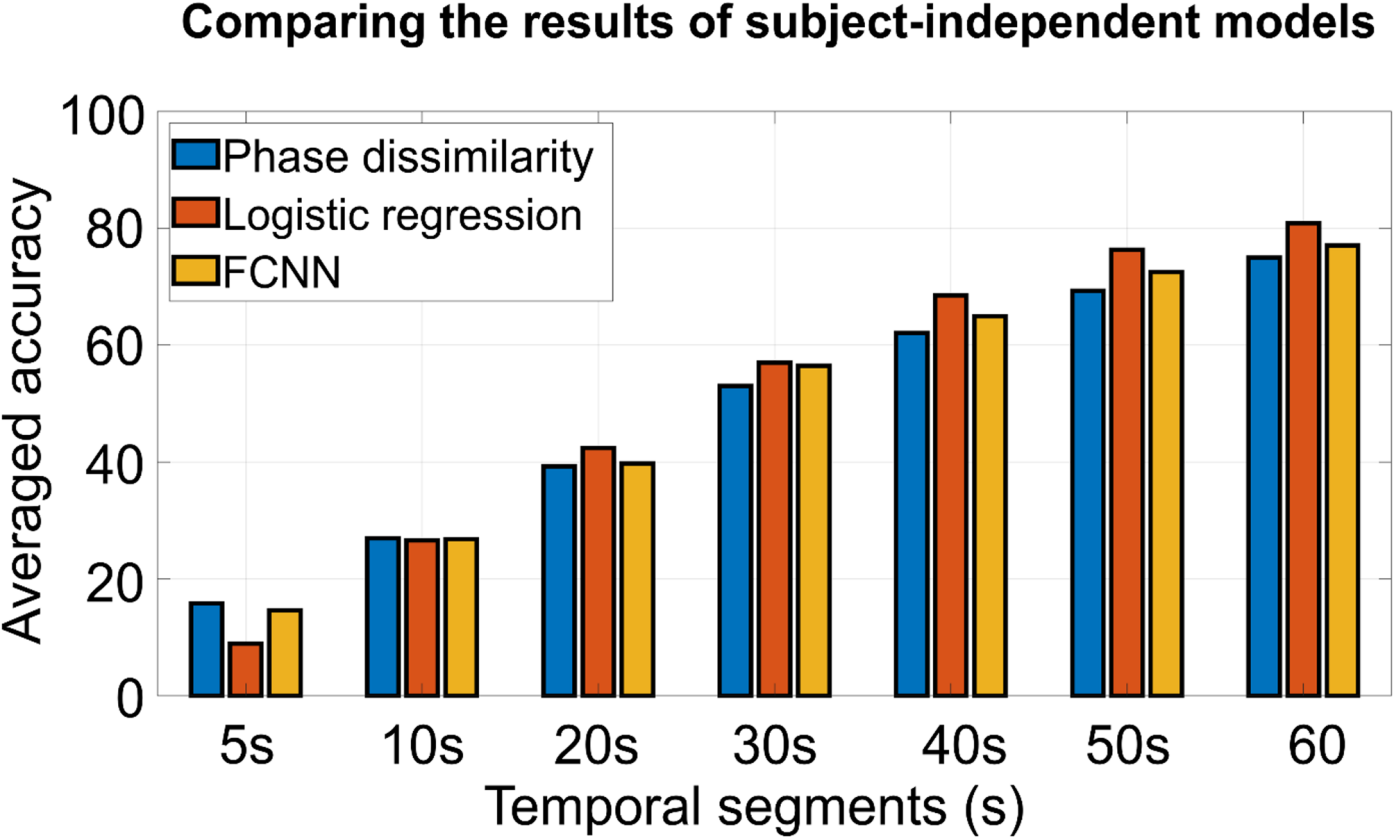
Comparison of average classification accuracy across subjects for three subject-independent models. The blue bars represent the performance of the subject-independent phase dissimilarity model. The red bars correspond to the subject-independent logistic regression classifier, and the orange bars denote the accuracy of the fully connected neural network (FCNN). The accuracies were averaged across all test subjects in all models.

To enhance the classification accuracy of the subject-independent phase dissimilarity model, we implemented two additional models: a subject-independent logistic regression classifier and an FCNN. Figure 6 presents the average classification accuracy of these three subject-independent models across test subjects for seven different EEG segment durations. When using the full one-minute EEG segment, the logistic regression model achieved an average accuracy of 80.84%, and the FCNN achieved 77.01%, both outperforming the phase dissimilarity model, which reached 74.94%. As shown in Fig. 6, reducing the EEG segment duration from one minute to 20 s still resulted in the logistic regression model outperforming the FCNN, and the FCNN outperforming the phase dissimilarity model. However, when the EEG segment duration was further reduced to 10–5 s, the phase dissimilarity model outperformed both machine learning and deep learning models. Specifically, with 10 s of EEG segments, the phase dissimilarity model achieved an accuracy of 27.01%, compared to 26.65% for logistic regression and 26.83% for the FCNN. With 5 s of EEG segments, the phase dissimilarity model reached 15.86%, while logistic regression and FCNN achieved 8.91% and 14.62%, respectively.

## Discussion

### Consistent cross-subject phase patterns in the delta and theta band

The results from the phase dissimilarity analysis indicate significant entrainments in delta ([1–4] Hz) and theta ([4–8] Hz) frequency bands, particularly in the bilateral frontal and frontocentral electrodes. The frontal and frontocentral electrodes used in our analysis (e.g., Fp1, Fp2, F3, F1, Fz, F2, F4, FC5, FC3, FC1, FCz, FC2, FC4, and FC6) overlie cortical regions involved in speech segmentation, temporal prediction, and attentional modulation—such as the inferior frontal gyrus, premotor cortex, and medial prefrontal areas^4,23,24^. These areas are known to support low-frequency phase entrainment to speech rhythms and have been implicated in speech intelligibility and cross-trial phase alignment^5^. Consistent with this, prior research has shown that EEG responses recorded from frontal and frontocentral regions reliably track the speech envelope and syntactic structure during naturalistic listening^25,26^. Collectively, these findings suggest that the brain regions corresponding to the significant electrode sites contribute to higher-order auditory processing essential for understanding continuous and meaningful speech.

The results from the phase dissimilarity analysis indicate significant entrainments in delta ([1–4] Hz) and theta ([4–8] Hz) frequency bands, particularly in the bilateral frontal and frontocentral electrodes. The frontal and frontocentral electrodes used in our analysis (e.g., Fp1, Fp2, F3, F1, Fz, F2, F4, FC5, FC3, FC1, FCz, FC2, FC4, and FC6) overlie cortical regions involved in speech segmentation, temporal prediction, and attentional modulation—such as the inferior frontal gyrus, premotor cortex, and medial prefrontal areas^4,23,24^. These areas are known to support low-frequency phase entrainment to speech rhythms and have been implicated in speech intelligibility and cross-trial phase alignment^5^. Consistent with this, prior research has shown that EEG responses recorded from frontal and frontocentral regions reliably track the speech envelope and syntactic structure during naturalistic listening^25,26^. Collectively, these findings suggest that the brain regions corresponding to the significant electrode sites contribute to higher-order auditory processing essential for understanding continuous and meaningful speech.Previous studies have explored the distinct roles of cortical speech tracking in the delta and theta bands for speech processing^27^. Findings demonstrate that cortical oscillations below 8 Hz are crucial for speech comprehension and intelligibility^28^. Generally, the delta band (1–4 Hz) is associated with processing words, phrases, and sentences, while the theta band (4–8 Hz) is linked to processing phonemes and syllables^11,29,30^. More specifically, delta-band cortical entrainment (1–4 Hz) remains robust in noisy environments, whereas theta-band entrainment (4–8 Hz) gradually decreases with increasing noise levels^23,31^. This suggests that delta-band tracking is closely tied to speech intelligibility in adverse listening conditions and remains stable unless significant changes in comprehension occur^32^. Conversely, theta-band tracking, while less robust as a standalone measure of intelligibility, plays an essential role in decoding syllabic acoustic features^6^. Our results indicate that cross-subject phase dissimilarity is higher in the delta band than in the theta band. This supports the notion that delta-band activity is strongly tied to the acoustical features of the speech envelope rather than higher-level cognitive processing^33^. Accordingly, we demonstrate that it is possible to distinguish among 22 audiobooks using a subject-independent model, as EEG phase patterns in the delta and theta bands are unique not only across trials within a subject (cross-trial) but also across trials between different subjects (cross-subject). The results of our classification algorithms support this claim: an average accuracy of 74.96%, well above the chance level of 10.61%, in identifying the correct audiobook from EEG responses demonstrates the viability of our approach using phase patterns from frontal and frontocentral electrodes.

Although classification performance correlates positively with the duration of EEG phase alignment (equivalent to the audiobook segment length), our findings show that even with only 5 s of input, the subject-independent models achieve accuracy above chance for all 17 subjects tested.

### Classification of EEG signals based on subject-independent models

These findings suggest that while subject-independent linear logistic regression and subject-independent non-linear FCNN models offer improved performance with longer EEG segments, the subject-independent phase dissimilarity model is more robust under limited temporal information. Moreover, the results of our subject-independent models surpass those of previous studies in several important aspects. First, unlike earlier research that relied on Magnetoencephalography (MEG)^5,20^we employed EEG devices. Although EEG signals generally have a lower signal-to-noise ratio (SNR) and are more susceptible to artifacts^34,35^they offer practical advantages for real-time applications such as speech detection due to their portability, affordability, and ease of use.

Second, our classification accuracy is significantly higher than those reported in prior studies. For consistent comparison, we employed Cohen’s kappa (*K*) as a performance metric^36^. Our models achieved classification accuracies of 74.96% (*K = 0.72*) for the phase dissimilarity model, 77.01% (*K = 0.74*) for the FCNN model, and 80.84% accuracy (*K = 0.79*) for the logistic regression model when classifying 22 audiobook stimuli. In contrast, Luo and colleagues reported less than 30% accuracy for classifying three audio sentences (*K = 0.00*) and six audiovisual movies (*K = 0.16*)^5,20^. Similarly, Ng et al. achieved 15.3% accuracy for 10 stimuli (*K = 0.06*)^21^Henry and Obleser reported approximately 60% for two stimuli (*K = 0.20*)^22^and Zuk et al. obtained less than 15% for 30 stimuli (*K = 0.12*)^14^. These comparisons underscore the superiority of our approach in terms of both raw accuracy and agreement measured by *K*.

Third, our methodology leverages transfer learning for subject-independent classification. That is, we classify the EEG signals of novel subjects using models trained on data from entirely different individuals, without requiring subject-specific calibration. This demonstrates not only the within-subject consistency of EEG phase patterns but also strong cross-subject generalizability.

Fourth, we use only a fixed number of constant frontal and frontocentral electrodes across all subjects for classification. In contrast, previous studies required a subject-specific set of electrodes^5^. This highlights the efficiency and scalability of our method, offering a generalized and streamlined approach to EEG-based speech decoding.

### Positive correlation between the accuracy and the duration of cross-subject EEG phase alignment

The results demonstrate a positive correlation between classification accuracy and cross-subject EEG phase alignment duration. Specifically, our findings indicate that shorter EEG segments, corresponding to shorter audiobook segments, result in lower classification accuracy. However, longer EEG segments lead to improved performance. This trend arises from longer EEG segments capturing more comprehensive temporal information, which enhances the identification of audiobook-specific phase patterns. These phase patterns are closely tied to acoustic events, such as speech onsets, that drive cross-subject phase synchronization in EEG signals^13,37,38^. Therefore, longer phase alignment intervals facilitate more robust detection of these discriminative patterns, thereby increasing classification accuracy.

### Positive correlation between the accuracy and number of cross-subject EEG phase alignments

It is expected that a subject-independent model will not outperform a subject-specific model when both are trained on only a single or identical template. However, the cross-subject EEG phase tracking shows high consistency because the EEG phase aligns strongly with the acoustic features of the stimuli. Consequently, increasing the number of cross-subject EEG phase alignments (i.e., templates) enhances the classification accuracy of the subject-independent model. As illustrated in Fig. 4, the subject-independent model requires at least nine templates, *on average*, to exceed the accuracy of the subject-specific model, regardless of the duration of cross-subject EEG phase alignments. This improvement is attributable to the enhanced signal-to-noise ratio obtained by averaging more templates. Moreover, Fig. 5 reveals a negative correlation between the duration of EEG segments and the number of templates required: shorter segments demand more templates, whereas longer segments require fewer templates to ensure that the subject-independent model achieves greater accuracy than the subject-specific model for *every subject*. In other words, if an equal number of templates is used, the model becomes less robust in classifying shorter EEG segments compared to the longer segments.

The findings of this study have several important implications. By demonstrating that EEG phase patterns in response to speech are consistent across individuals, we provide evidence for a shared neural encoding of acoustic information that can be utilized in subject-independent decoding models. This is a crucial step toward building more generalizable and scalable EEG-based applications, such as brain–computer interfaces, neuroadaptive hearing aids, and cognitive load monitoring systems that do not require individual calibration. Additionally, the ability of speech prediction of a listener supports the idea of stable neural entrainment mechanisms to speech rhythm, which could enhance our understanding of auditory processing and language comprehension^25,39^. Our methodology offers a reproducible approach for investigating shared neural responses across individuals, particularly in future studies utilizing naturalistic stimuli and large-scale datasets.

Our results demonstrate the existence of cross-subject EEG phase alignment through the high classification accuracy of a subject-independent EEG model; however, several limitations should be acknowledged. Firstly, it is noteworthy that English was the second language for all listeners, who had a strong background in speaking. It would be interesting to collect data from native speakers and compare the accuracy with participants for whom English is a second language. Additionally, we employed ICA as a robust signal processing technique to clean and remove artifacts. However, ICA is primarily suited for offline analysis, as a substantial number of samples (25 times the number of electrodes squared) are required for optimal performance^40^. Performing real-time classification, especially for shorter segments, poses challenges due to the inherently noisy nature of EEG signals. These signals are susceptible to artifacts such as eye movements and muscle activity, which can negatively impact classification accuracy in real-time applications.

## Materials and methods

### Participants

Participants consisted of 17 healthy right-handed non-native English speakers (five females) between 22 and 33 years of age (mean = 25.76, SD = 3.32) with normal hearing, normal or corrected-to-normal vision, and no neurological/psychiatric disorders, and no history of hearing impairment (according to self-reports). All of them were unfamiliar with the stories employed in this study, and English was their second language.

The Ethics Committee at the Technical University of Munich approved the experimental research protocol (reference number 686/21 S-KK). All individual participants signed a consent form before the experiment and received moderate monetary compensation for their participation.

### Stimuli

This study employs 22 English audiobooks from 22 different literature works, read by various male speakers. The study also used one extra audiobook to familiarize participants with the experimental protocol (pilot audiobook). All audios were normalized to 20 dB with a sampling frequency of 44.1 kHz. Each audiobook lasted between 65 and 73 s. The audio resources are mentioned in Table 2.

**Table 2:** The name of audiobooks used in this study.

#	Name of book	Writer	Time (s)	Link for the audios
Pilot	The Adventure of the ‘Western Star’	Agatha Christie	68	https://bookaudio.online/719-the-adventure-of-the-western-star.html
1	The Old Man and the Sea	Ernest Hemingway	70	https://bookaudio.online/78-ernest-hemingway-the-old-man-and-the-sea.html
2	The Legend of Sleepy Hollow	Washington Irving	68	https://librivox.org/the-legend-of-sleepy-hollow-by-washington-irving/
3	The Talisman	Stephen King, Peter Straub	65	https://bookaudio.online/586-the-talisman.html
4	In Dubious Battle	John Steinbeck	69	https://bookaudio.online/179-john-steinbeck-in-dubious-battle.html
5	Martin Eden	Jack London	67	https://bookaudio.online/236-jack-london-martin-eden.html
6	Being Mortal: Medicine and What Matters in the End	Atul Gawande	67	https://bookaudio.online/317-atul-gawande-being-mortal-medicine-and-what-matters-in-the-end.html
7	Private London	James Patterson, Mark Pearson	65	https://bookaudio.online/417-private-london.html
8	Steve Jobs	Walter Isaacson	69	https://bookaudio.online/464-steve-jobs.html
9	Blueeyedboy	Joanne Harris	66	https://bookaudio.online/377-joanne-harris-blueeyedboy.html
10	Honolulu	William Somerset Maugham	69	https://bookaudio.online/85-william-somerset-maugham-honolulu.html
11	Across the River and into the Trees	Ernest Hemingway	68	https://bookaudio.online/62-ernest-hemingway-across-the-river-and-into-the-trees.html
12	Holiday Task	Saki	69	https://bookaudio.online/523-holiday-task.html
13	Interlopers	Saki	73	https://bookaudio.online/528-interlopers.html
14	Straight	Dick Francis	70	https://bookaudio.online/273-dick-francis-straight.html
15	Dead Man’s Time	Peter James	65	https://bookaudio.online/397-peter-james-dead-mans-time.html
16	Goodbye, Jack	Jack London	66	https://bookaudio.online/244-jack-london-goodbye-jack.html
17	The Looking Glass War	John le Carré	65	https://bookaudio.online/392-john-le-carr%C3%A9-the-looking-glass-war.html
18	The Amber Room	Steve Berry	68	https://bookaudio.online/606-the-amber-room.html
19	Cross Country	James Patterson	70	https://bookaudio.online/426-cross-country.html
20	Forfeit	Dick Francis	68	https://bookaudio.online/290-dick-francis-forfeit.html
21	The Call of The Wild	Jack London	68	https://bookaudio.online/262-jack-london-the-call-of-the-wild.html
22	Dead Man’s Footsteps	Peter James	68	https://bookaudio.online/401-peter-james-dead-mans-footsteps.html

### Protocol

Participants listened to each audiobook using Sennheiser Momentum2 headphones (equipped with a 3.5-mm jack plug) while seated comfortably and facing a monitor. Each participant completed a trial session by listening to an audiobook different from those used in the main study to become familiar with the experimental procedure. Once participants indicated their readiness to commence, a prompt appeared, and the experiment began four seconds after the participant pressed a button. During the experiment, participants listened to each of the 22 audiobooks listed in Table 2 three times. Throughout the listening sessions, participants were instructed to focus on a fixation cross displayed on the screen and to minimize any movements, such as chewing, hand or leg movements, and excessive blinking. After each listening session, participants answered one or two questions about the audiobook to ensure attention to the audio content. After 12 trials, each consisting of three listening sessions to four different audiobooks, the participants were allowed to take breaks to prevent fatigue and discomfort, and they could resume the experiment at their convenience.

### EEG recording and preprocessing

EEG data were collected using a Brain Products ActiChamp amplifier equipped with 59 gel-based electrodes, positioned according to the 10–10 international system. The ground electrode was positioned 1.5 cm in front of the frontocentral area, matching electrode Fpz’s location. As a reference, two electrodes (TP9-TP10) were placed behind the ears (linked mastoids). Additionally, three electrodes were positioned on the center of the forehead and below the right and left outer canthi to record vertical and horizontal eye movements, known as electrooculograms (EOG). Participants were instructed to keep their heads still and refrain from chewing gum, mumbling to themselves, or making any other movement. The examiner closely monitored the recording process to identify faulty trials and artifacts. EEG signals were collected at a frequency sampling of 1000 Hz to achieve higher temporal resolution. All electrode impedance values were kept below 50 K throughout the experiment to provide a high signal-to-noise ratio. During the recording, the data was sent to a different recording PC (Intel^®^ CoreTM i5 CPU 750@2.67 GHz) via USB^41,42^.

All analyses were performed in the Matlab environment using the EEGLAB toolbox^43^ (https://sccn.ucsd.edu/eeglab/index.php). The continuous raw data were passed through a non-causal and zero-phase (forward-reverse) bandpass FIR (i.e., finite impulse response) filter with a cut-off frequency (−6 dB) of 0.25 Hz and 60.25 Hz (performing 6601 points bandpass filtering with a transition bandwidth of 0.5 Hz; passband edge: [0.5 60] Hz). Subsequently, a non-causal and zero-phase notch filter was applied at 50 Hz with a cut-off frequency of 48 Hz and 51 Hz (−6 dB) to remove line noise (performing 1651-point band stop (notch) filtering with a transition bandwidth of 2 Hz; passband edge: [48 52] Hz). Then, raw data were resampled from 1000 Hz to 500 Hz. The raw data were segmented into stimulus time-locked 66 epochs (22 audiobooks * 3 repetitions) ranging from − 3 s to 65 s. Then, after careful monitoring of all epochs, bad electrodes corresponding to bad epochs were detected and interpolated with the spherical method (interpolated electrodes across the participants: mean: 2.18, SD: 2.21). All epoch data was subjected to independent component analysis (ICA) once to identify artifacts (e.g., eye blink, eye movement, and muscular activity) that the filter processes could not remove. Because of its higher performance, the SOBI-ICA, as implemented in EEGLAB, was chosen for the purpose of this study. Since SOBI is a second-order blind source separation technique, it eliminates EOG and electromyography artifacts more accurately and retains more brain activity than higher-order statistical techniques such as INFOMAX, FastICA, and Jade^44,45^. The suspicious ICA components were eliminated after careful monitoring (mean of removed components per subject: 46.29, SD: 4.93). It is important to mention that our paradigm involved continuous listening to one-minute-long auditory stimuli, which naturally increases the likelihood of various artifacts (e.g., muscle activity, eye movements, or slow drifts) distributed over different time points. Thus, the number of removed ICA components is relatively high compared to typical EEG studies. As a result, we obtained 66 clean trials (22 audiobooks * 3 repetitions), each containing 59 electrodes ranging from − 3 s to 65 s.

### Cross-subject phase dissimilarity

Phase dissimilarity is a widely recognized method^5,14,16,33^ to evaluate the phase consistency of EEG trials corresponding to one audiobook. Cross-subject phase dissimilarity was computed as the difference in cross-trial phase consistency between trials of all subjects elicited by the same stimulus (within-group) and trials of all subjects from different stimuli (across-group). This phase-only metric captures the extent to which the phase of neural oscillations is reliably aligned across repeated exposures to the same stimulus (see Fig. 1A). Initially, all 66 trials underwent resampling from 500 Hz to 200 Hz. Following previous work^5,33^within-group signals were derived from all trials from all subjects related to the same speech stimulus. This resulted in 22 within-groups, each containing 51 trials (3 repetitions × 17 subjects). The concatenation of trials across subjects was performed to examine the cross-subject EEG phase tracking. To create a comparison baseline, all 1,122 trials (22 stimuli * three repetitions * 17 subjects) were randomly shuffled and reassigned to form 22 new groups, each containing 51 trials. These randomly assembled groups are referred to as across-group signals. A total of 1,000 different across-groups were generated from the original dataset to ensure a robust statistical comparison. Time-frequency responses (TFRs), implemented in the FieldTrip toolbox^46^ (http://www.fieldtriptoolbox.org/), were then conducted for each trial in the within-groups and the 1000 across-groups, using the mtmconvol method with a Hanning taper and three cycles (i.e., time_window = 3/frequency), with a temporal resolution of 0.2 s for across a time range from 1.4 s to 60 s and a frequency range from 1 Hz to 45 Hz to ensure covering frequency bands from delta to gamma and avoiding the first second (0–1 s) due to the event-related potential (ERP) effect. Thus, a 3D matrix was generated for each trial, consisting of 59 electrodes, 56 frequency bins, and 292 time points. The cross-subject phase coherence values were then computed, as^5^:

$$\:{CPhase}_{kij}=\:\frac{\sqrt{{\left(\sum\:_{n=1}^{N}\text{c}\text{o}\text{s}\left({\theta\:}_{knij}\right)\right)}^{2}+{\left(\sum\:_{n=1}^{N}\text{s}\text{i}\text{n}\left({\theta\:}_{knij}\right)\right)}^{2}}}{N}$$

where $\:{\theta\:}_{knij}$ is the phase for frequency bin *i* and time bin *j* in trial *n* and group *k* (*k* = 22 stimuli) with $\:N=3*17$ (three repetitions * 17 subjects) in this study. Cphase is a number between 0 and 1. A larger Cphase value indicates strong cross-subject phase coherence. The calculated cross-trial Cphase was compared separately between the 22 within-group signals and each of the 1000 across-group signals. Then, the dissimilarity function for each frequency bin *i* was measured as follows:

$$\:{Dissimilarity\_phase}_{i}=\:\frac{\sum\:_{k=1}^{K}(\frac{\sum\:_{j=1}^{J}\left({CPhase}_{kij,within}\right)}{J}-\frac{\sum\:_{j=1}^{J}\left({CPhase}_{kij,across}\right)}{J})}{K}$$

where $\:J=292\:$(time points) and K = 22 (number of audiobooks). A higher value of phase dissimilarity at frequency bin *i* indicates that the stimulus elicits temporally consistent phase responses from trial to trial in EEG signals at this frequency bin. Dissimilarity phase values were computed for each of the 59 EEG electrodes.

### Statistical analysis

To address the multiple comparisons problem (MCP), which arises from the large number of electrodes and frequency bins and can inflate the family-wise error rate (FWER)^47^we employed a one-tailed non-parametric cluster-based permutation test. This method reduces the likelihood of Type I errors by using a data-driven clustering approach that accounts for the spatial and spectral structure of the data. Specifically, we constructed a null distribution using 1,000 surrogates across-group datasets (CPhase_across), which were generated by randomly shuffling the original within-group trials. Significant clusters were then identified by comparing the observed data against this null distribution. The alpha level was set at 0.025, meaning that an electrode–frequency bin was considered significant if the associated p-value was below this threshold (*p* < 0.025)^48^. To visualize the phase dissimilarity in the frequency domain, mean dissimilarity phase values were calculated across all 59 electrodes (see Fig. 1B). The dissimilarity phase values were then averaged across all 17 subjects and across all significant frequency bins (i.e., 1–8 Hz) to identify the electrodes showing the highest dissimilarity. The most significant electrodes, corresponding to the maximum dissimilarity phase values, are illustrated in Fig. 1C.

### Cross-subject correlation coefficient

As an alternative to the phase dissimilarity analysis, we also computed cross-subject correlation coefficients to assess EEG phase alignment by evaluating the linearity of EEG trials across subjects in response to a specific audiobook. To do this, we first computed TFRs using the same procedure described in the phase dissimilarity section. Specifically, we employed the mtmconvol method with a Hanning taper and three cycles, at a temporal resolution of 0.05 s, over the time range from 1.4 s to 60 s. Analyses were performed for two frequency bands: the delta band (1–4 Hz) and the theta band (4–8 Hz).

The resulting dataset comprised a five-dimensional matrix with the following dimensions: 59 electrodes, 2 frequency bands, 1165 time points, 22 stimuli, and 51 EEG trials (3 repetitions × 17 subjects). For simplicity, data were averaged across electrodes. Then, for each frequency band and stimulus, we computed the cross-correlation coefficient for all possible pairs of the 51 EEG phase trials, resulting in 1,275 unique pairings. Cross-correlation was computed across time lags ranging from − 1.5 s to + 1.5 s. The absolute values of these cross-correlation coefficients were then averaged across all stimulus and trial pairs to produce a two-dimensional correlation matrix with dimensions corresponding to frequency band (2) and time points (1165).

To conduct statistical analysis on the identified correlations, we assumed the null hypothesis of no correlation between EEG phase trials across subjects. To test this, we created a surrogate dataset by randomly shuffling data across both trials and stimuli. We then conducted the same correlation analysis on this surrogate data to generate a null distribution of correlation matrices. This process was repeated 100 times to estimate the range of correlation values anticipated under the null hypothesis.

To correct for MCP, we conducted a one-tailed, non-parametric, cluster-based permutation test with a significant threshold of *P* < 0.01. A time lag sample was considered significant if the corresponding P-value fell below this threshold (*P* < 0.01).

### Subject-specific dissimilarity model

We built on the methodology of a previous work^5^ to classify new single-trial EEG responses to predict which audiobooks the participant listened to. We employed a nearest-template classification approach to identify which audiobook a subject was listening to, based on EEG phase information. Rather than using a traditional supervised learning algorithm (e.g., SVM, LDA, or logistic regression), we adopted a template-matching method grounded in cross-trial phase coherence (CPhase), which is particularly well-suited for evaluating similarity in phase space.

For each classification iteration, we randomly selected one EEG trial for each of the 22 audiobooks to serve as a template, resulting in 22 templates per iteration. Each template was a three-dimensional representation of EEG phase information, consisting of 292 time points × 20 electrodes × 15 frequency bins. The remaining 44 trials (2 trials per audiobook) served as test data. For each test trial, we computed its CPhase with each of the 22 templates. This CPhase measure was then averaged across time (temporal), frequency (spectral), and electrodes (spatial) to yield a single similarity value per template. The predicted label for the test trial was the audiobook whose template yielded the highest CPhase value.

To ensure robustness and generalizability, we repeated this classification process 200 times, each time with a new random selection of template trials. This permutation-based evaluation approximates a cross-validation framework and is particularly effective when the number of trials per condition is limited. The classification accuracy was averaged over these 200 iterations, providing a stable estimate of model performance. Given 22 audiobook classes and 44 test trials, the chance-level classification accuracy was not estimated naively as 1/22 (≈ 4.55%), but rather determined using the binomial inverse cumulative distribution function to account^49^ for statistical significance at the 0.01 level. Specifically, chance-level accuracy was computed as:

$$\:Chance - level_{{accuracy}} = \:{\text{min}}\left\{ {k\: \in \:\left\{ {0,\:1,\: \ldots \:,\:n} \right\}:\:\sum {\:_{{i = 0}}^{k} } \left( {\begin{array}{*{20}c} n \\ {\:i} \\ \end{array} } \right)p_{{succ}} ^{i} \:\left( {1 - p_{{succ}} } \right)^{{n - i}} \ge \:p} \right\} \times \:\frac{{100}}{n}$$

$\:\text{C}\text{h}\text{a}\text{n}\text{c}\text{e}-\text{l}\text{e}\text{v}\text{e}{\text{l}}_{\text{a}\text{c}\text{c}\text{u}\text{r}\text{a}\text{c}\text{y}}\:$is 13.64%, where $\:\text{n}=44$ is the number of test trials, $\:{p}_{succ}=\frac{1}{22}$ is the probability of success under random guessing, $\:p=1-\:\alpha\:=0.99$ represents the cumulative probability threshold. This formulation allows us to define a statistically meaningful baseline for evaluating model performance beyond chance.

### Subject-independent dissimilarity model

In this model, templates were constructed by averaging the EEG phase patterns of all subjects except the test subject. Specifically, we used data from 16 participants, each with 3 repetitions across 22 audiobooks, and averaged the trials across both repetitions and subjects. The EEG trials from the held-out subject (66 trials in total) were used as test data. The chance-level classification accuracy for this setup was calculated to be 10.61%, based on the binomial inverse cumulative distribution function (with parameters: $\:\text{n}=66$, $\:{p}_{succ}=\frac{1}{22}$, and $\:p=1-\:\alpha\:=0.99$).

To ensure a fair comparison with the subject-specific model, we used the same set of the top twenty most significant electrodes. The remaining classification procedure, i.e., computing CPhase between each test trial and the average templates, was identical to the subject-specific model. However, unlike the subject-specific approach, no permutation was required here, since each subject had a fixed set of templates derived from independent data. This method has the distinct advantage of enabling EEG response classification for a new subject without requiring any training data from that subject.

### Random classifier

To assess whether phase-based coherence provides meaningful classification value, we implemented random classifiers as baselines for both the subject-specific and subject-independent models. This baseline simulates classification performance in the absence of any systematic relationship between EEG phase patterns and audiobook identity. We maintained the same number of classes (22) and the same number of test trials. For each iteration, class labels were randomly assigned to the test trials, thereby disrupting any true correspondence between EEG data and stimulus identity. This random labeling procedure was repeated 200 times to generate null distributions of classification accuracies. The mean accuracy across these 200 iterations was then computed, providing a reference point against which the actual model performance could be meaningfully compared.

logistic regression model

### Subject-independent logistic regression model

To improve classification accuracy and benchmark our simple model──based on nearest-template classification──against other machine learning approaches, we implemented a logistic regression classifier as a representative linear model. Time–frequency representations of the EEG phase, as described in the Phase Dissimilarity section, were used for feature extraction.

For each subject, we trained the model using data from the remaining 16 subjects and optimized the hyperparameters during validation. This resulted in a total of 1,056 training and validation trials (22 stimuli × three repetitions × 16 subjects). From the EEG phase data, we extracted the cosine (real part) and sine (imaginary part) components and averaged them across electrodes to reduce dimensionality.

We employed five-fold cross-validation to optimize the regularization parameter (i.e., lambda and kernel size) for the logistic regression classifier. For template construction, 132 templates were created by averaging across trained subjects for each repetition, stimulus, and each phase component (i.e., cosine and sine). We then computed the correlation coefficients in the time domain between each training and test trial and the 132 templates. The resulting correlations were averaged across frequency, repetition, and phase component dimensions, yielding 22 features per trial.

Additionally, we repeated the correlation analysis for the first and second halves of each EEG trial, resulting in a total of 66 feature vectors per trial. For the classification task, we adopted the One-vs-All (OvA) strategy to handle the 22-class classification problem by converting it into a series of binary classification tasks. As with the phase dissimilarity models, this classification was repeated across different EEG trial durations.

### Subject-independent fully connected neural network (FCNN) model

To enhance classification performance and to compare our nearest-template model with an alternative machine learning approach (i.e., logistic regression), we implemented a non-linear FCNN. For each test subject, the FCNN was trained using data from the remaining 16 subjects. An 8-fold cross-validation scheme was applied within the training set (i.e., 14 subjects for training and 2 for validation) to optimize the network weights, while the test subject was held out for final evaluation. To address the limitation of training data size, we employed a data restructuring strategy wherein each electrode’s signal was treated as an independent trial. This reshaped the data from [time × frequency × electrodes × trials] to [time × frequency × (electrodes × trials)], effectively increasing the number of training samples.

Similar to the logistic regression model, we used correlation coefficients as features. However, in contrast to the logistic model, which extracted features from the entire and both the first and second halves of EEG trials, the FCNN used correlation features computed over the entire trial duration, resulting in 22 feature vectors per sample. The FCNN architecture consisted of an input layer matching the feature dimensionality, followed by two fully connected layers with 44 units each. Each dense layer was followed by batch normalization, an exponential linear unit (ELU) activation function with α = 1.0, and a dropout layer with a rate of 0.2 to mitigate overfitting. The output layer was a fully connected layer with 22 units corresponding to the EEG classes (i.e., stimuli), followed by a softmax activation for multi-class probability distribution. The model was trained using stochastic gradient descent with momentum (SGDM) over 50 epochs, with a mini-batch size of 64 and a learning rate of 0.01. Validation was performed every 50 iterations, and the best model was selected using early stopping with a patience of 15 validation steps.

## Data Availability

Data and code availability: The data and codes supporting the findings of this study will be openly available in https://figshare.com/s/8996724ddd0148d7626d at DOI: 10.6084/m9.figshare.24920325 AND https://figshare.com/s/3f4f060d615bf2f75a8e at DOI: 10.6084/m9.figshare.24916683.

## Abbreviations

EEG: Electroencephalograph
MEG: Magnetoencephalography
ICA: Independent component analysis
INTERACT: Brain-To-Sound Computer Interfaces: The effect of having prior knowledge on neural tracking of speech/music
